# Effectiveness and tolerability of perampanel monotherapy in children with newly diagnosed focal epilepsy

**DOI:** 10.3389/fneur.2023.1144759

**Published:** 2023-05-24

**Authors:** Fen Zhao, Ying Ren, Guifu Geng, Tong Zhang, Wandong Hu, Huan Zhang, Ruifeng Jin, Jianguo Shi, Zaifen Gao, Hongwei Zhang, Yong Liu

**Affiliations:** ^1^Epilepsy Center, Children's Hospital Affiliated to Shandong University, Jinan, China; ^2^Epilepsy Center, Jinan Children's Hospital, Jinan, China

**Keywords:** perampanel, focal epilepsy, children, tolerability, effectiveness

## Abstract

**Objective:**

To examine the clinical effectiveness and tolerability of perampanel (PER) as initial monotherapy in pediatric patients with newly diagnosed focal epilepsy.

**Methods:**

A retrospective analysis was conducted on 62 children with newly diagnosed focal epilepsy who were treated with PER at the Epilepsy Center of Jinan Children's Hospital from July 2021 to July 2022. The treatment status, prognosis, and adverse reactions were followed up for a minimum of 6 months after the initiation of PER monotherapy. The effectiveness of the patients was estimated by the PER effective rate at 3-, 6-, and 12-month follow-up evaluations and adverse reactions were also recorded. The effective rates of PER in different etiologies and epilepsy syndromes were also statistically analyzed.

**Results:**

The effective rates of PER treatment at the different time points of evaluation were 88.7% (3 months), 79.1% (6 months), and 80.4% (12 months). With PER treatment, seizure freedom varied over time, with 61.3%, 71.0%, and 71.7% of patients at the 3-, 6-, and 12-month follow-ups, respectively. Among the etiologies of epilepsy, the effective rates of genetic etiology, structural etiology, and unknown etiology were generally above 50% at the 3-, 6-, and 12-month follow-ups. Among the epilepsy syndromes, the categories with higher treatment efficacy were self-limiting epilepsy with centrotemporal spikes (SeLECTs), self-limited epilepsy with autonomic seizures (SeLEAS), and childhood occipital visual epilepsy (COVE), with an effective rate of above 80%. Adverse events were documented in 22 patients (35.5%), but they were mild and tolerable. The most common adverse events comprised irritability, drowsiness, dizziness, and increased appetite.

**Conclusion:**

PER has favorable effectiveness and tolerability as initial monotherapy for children with newly diagnosed focal epilepsy, which could be a potential option for long-term medication in the treatment of focal epilepsy in children. The current study provided potential evidence for PER as initial monotherapy in children with focal epilepsy in clinical practice.

## Introduction

Epilepsy is a common pediatric neurological condition characterized by an enduring predisposition to cause epileptic seizures, with an estimated worldwide prevalence of 10.5 million children aged under 15 years, representing approximately 25% of the global epilepsy population (1). Focal epilepsy is the most common type of epilepsy, prevalent in 45% of children (2), where seizures arise from epileptogenic zones within the cerebral cortex, most commonly in the temporal or frontal lobe (3). According to the guidelines provided by the International League Against Epilepsy (ILAE), when indicated, the monotherapy of anti-seizure medications (ASMs) was the first recommendation regarding focal epilepsy (4). Certainly, ASMs should not be prescribed for self-limited focal epilepsies with a lower seizure frequency (once a year or less). With each subsequent ASM regimen trialed, the probability of achieving seizure freedom diminished substantially; most patients who gain seizure control with ASMs do so with the first or second prescribed dose (5). Previous studies have shown that ~70% of patients achieve seizure freedom by conventional ASMs (6); however, approximately one-third of patients with focal-onset seizures still have uncontrolled seizures and develop refractory epilepsy (7). In addition, children with persistent focal seizures have a considerable risk of cognitive impairment, behavioral problems, and an overall compromised quality of life (8). Therefore, it is critical to select an effective ASM as initial monotherapy for children with newly diagnosed focal epilepsy to realize the best possible therapeutic outcomes early.

Perampanel (PER), as a novel ASM in recent years, is the first non-competitive antagonist that selectively acts on α-amino-3-hydroxy-5-methyl-4-isoxazole propionic acid (AMPA)-type glutamate receptors at the postsynaptic level (9, 10). It was first approved by the Food and Drug Administration (FDA) and the European Medicines Agency (EMA) in 2012 for focal-onset seizures with or without focal to bilateral tonic-clonic evolution (9). In China, it was approved in 2019 as an add-on treatment of focal epilepsy (with or without secondarily generalized seizures) at 12 years of age and older and in July 2021 for monotherapy use for focal epilepsy (with or without secondarily generalized seizures) at 4 years of age and older (10). Additionally, PER has a once-daily dosing schedule that supports children's adherence (11). In previous studies, the effectiveness and tolerability of PER as an add-on or adjunctive treatment for focal epilepsy in children have been demonstrated (12–14). However, the existing data on the effectiveness and tolerability of PER as an initial monotherapy for children with newly diagnosed focal epilepsy are still limited. Therefore, the current study aimed to examine the clinical effectiveness and tolerability of PER as an initial monotherapy in pediatric patients with newly diagnosed focal epilepsy to provide an initial insight into and data support for the clinical practice of PER monotherapy in children with new focal-onset epilepsy.

## Methods

### Patients

A retrospective analysis was performed on 62 children with epilepsy who received PER as monotherapy in the Epilepsy Center at Jinan Children's Hospital from July 2021 to July 2022. The inclusion criteria were as follows: (1) children aged ≤18 years; (2) patients who met the 2017 ILAE Classification of Epileptic Seizures for newly diagnosed epilepsy with focal-onset seizures (4); (3) patients never receiving prior ASMs; and (4) patients who agreed and signed the emotional consent form and were treated with PER monotherapy. The exclusion criteria were (1) patients over 18 years of age and older; (2) patients without newly diagnosed focal epilepsy; (3) patients not treated with only PER; (4) patients with severe other diseases affecting follow-up; and (5) patients who disagreed to participate in this study. The diagnosis was established by clinical history and an electroencephalogram (EEG), which were consistent with focal-onset seizures. Patients with a normal EEG could be included, provided they met the diagnostic criteria according to their clinical history.

**Figure 1:**
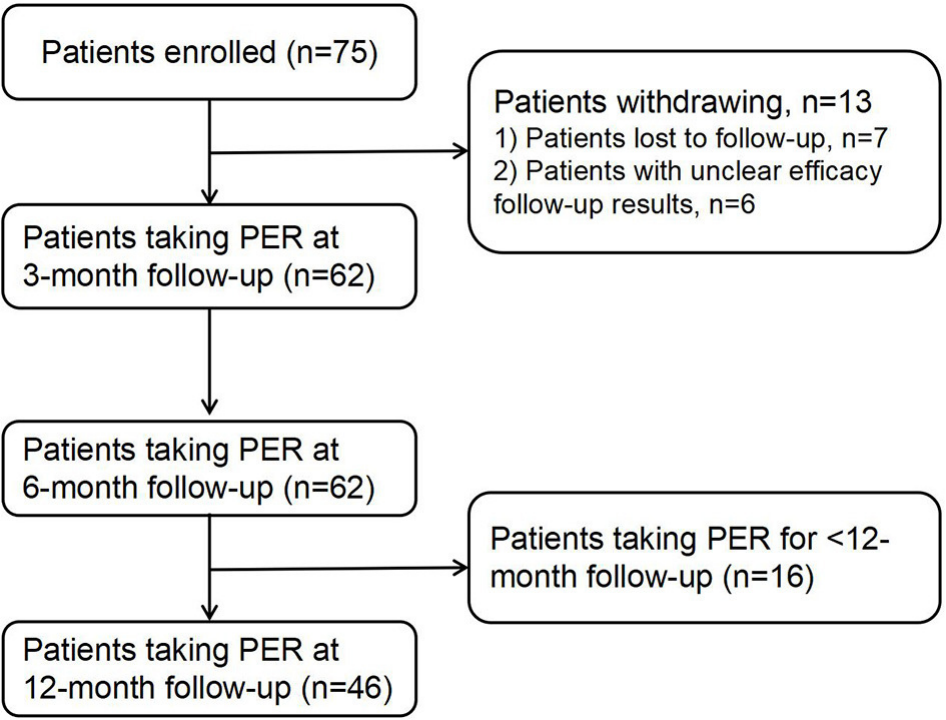
Flowchart of 62, 62, and 46 patients at 3-, 6-, and 12-month follow-ups.

The enrolled patients were followed up via telephone, and their treatment status was recorded. Among the 75 follow-up patients, seven were lost to follow-up and six follow-ups had unclear results; thus, 62 patients were included in the study. All the patients who completed the follow-up were followed up for more than 6 months, of which 46 were followed up for more than 12 months (Figure 1). The current research complied with the requirements of the World Medical Association Declaration of Helsinki, and all patients or caregivers signed the informed consent form. This study was approved by the Ethics Committee of Jinan Children's Hospital (approval number: QLET-IRB/P-2021088).

### Treatment methods and observation indicators

All patients diagnosed with focal epilepsy were treated with PER monotherapy after admission. For children aged below 4 years, the initial dose of PER was 0.5 mg/day; for the children aged 4–12 years, the initial dose of PER was 0.5, 1, and 2 mg/day according to the children's weight, that is, <20 kg, 20–30 kg, and > 30 kg, respectively; for the children aged above 12 years, the initial dose of PER was 2 mg/day. The dose of PER was increased by a maximum of 2 mg/day/week to a maximum of 8 mg/day. The maintenance dose depended on clinical effect and tolerance. Patients took the PER once a day before sleeping and did not stop or miss it during treatment. The treatment time was more than 6 months.

Patient data regarding some demographics, etiologies, seizure types, epilepsy syndromes, epilepsy duration before treatment with PER, a maintenance dosage of PER, and seizure frequency before and after treatment initiation were collected from medication records. The treatment status, prognosis, and drug-related adverse reactions of all patients were collected during administration and follow-up. Seizure frequency at baseline was defined as the mean of seizure counts in the 6 months prior to PER treatment. The treatment-effective response rate of epilepsy was evaluated as a reduction of ≥50% compared to baseline or seizure freedom at 3-, 6-, and 12-month follow-ups. The treatment-effective response rates of different etiologies and epilepsy syndromes were statistically analyzed. Seizure freedom was defined as having no seizures during the previous 3 months. Tolerability was assessed by the documentation of possible adverse reactions during treatment. Information regarding adverse reactions was recorded according to reports from the patients themselves or their caregivers.

### Statistical analysis

SPSS 26.0 software was used for data analysis. A Shapiro–Wilk test was used to test for sample distribution. The continuous data with a normal distribution were expressed as mean ± standard deviation (SD), while the data with a non-normal distribution were expressed as median (interquartile range), and the comparisons were examined using the Student's *t*-test and the Mann–Whitney test (non-parametric distribution). The categorical data were expressed as *n* (%). The comparison between the two groups was examined by chi-square analysis or Fisher's exact test. A *p*-value of <0.05 indicates that the comparison was statistically significant.

## Results

### Demographic and clinical information

A total of 62 patients were enrolled in the study, including 39 males and 23 females. The median onset age of starting PER treatment was 6.63 years (4.15, 9.00). The median weight was 22.50 (17.50, 34.13) kg, and the duration of epilepsy was 4.5 months (2.00, 12.25). There were 33 patients (56.5%) with genetic etiology, 14 (22.6%) with structural etiology, and the remaining 13 patients (20.9%) with unknown etiology. In epilepsy syndromes, 23 patients were diagnosed with self-limiting epilepsy with centrotemporal spikes (SeLECTs), two patients with sleep-related hypermotor epilepsy (SHE), six with childhood occipital visual epilepsy (COVE), four with self-limited epilepsy with autonomic seizures (SeLEAS), and 27 with an unclarified type of epilepsy. The median maintenance dose of PER was 4 mg/day (2.00, 4.00). Moreover, there was no statistical significance in the effectiveness between these variables at the 6-month follow-up (all *p*-values > 0.05), as shown in Table 1.

**Table 1:** General information of patients and comparison of effective rate of PER in different variables at 6 months of follow-up.

**Index**	**Total (*n* = 62)**	**Effective (*n* = 49)**	**Noneffective (*n* = 13)**	**p-value**
Age (year)	6.71 ± 3.01	7.07 ± 2.92	5.36 ± 3.10	>0.05
**Sex**				
Male	39 (62.9%)	30 (76.9%)	9 (23.1%)	>0.05
Female	23 (37.1%)	19 (82.6%)	4 (17.4%)	
Weight (kg)	22.50 (17.50, 34.13)	23.00 (17.75, 35.50)	21.00 (15.00, 25.25)	>0.05
Onset age of PER (year)	6.63 (4.15, 9.00)	7.00 (4.30, 9.00)	5.00 (2.88, 7.00)	>0.05
Duration of epilepsy (month)	4.50 (2.00, 12.25)	5.00 (3.00, 12.50)	3.00 (0.40, 10.00)	>0.05
Seizure frequency at baseline (monthly)	2.00 (1.00, 4.00)	2.00 (1.00, 4.00)	4.00 (1.00, 34.00)	>0.05
**Etiology**				
Genetic	35 (56.5%)	32 (91.4%)	3 (8.6%)	>0.05
Structural	14 (22.6%)	7 (50.0%)	7 (50.0%)	
Encephalomalacia foci	6 (9.7%)	4 (66.7%)	2 (33.3%)	
MCD	8 (12.9%)	3 (37.5%)	5 (62.5%)	
Unknown	13 (20.9%)	10 (76.9%)	3 (23.1%)	
**Epileptic syndrome**				
SeLECTs	23 (37.1%)	21 (91.3%)	2 (8.7%)	>0.05
COVE	6 (9.7%)	5 (83.3%)	1 (16.7%)	
SHE	2 (3.2%)	1 (50.0%)	1 (50.0%)	
SeLEAS	4 (6.5%)	4 (100%)	0	
Unknown	27 (43.5%)	18 (66.7%)	9 (33.3%)	
Maintenance dose of PER (mg/day)	4.00 (2.00, 4.00)	4.00 (2.00, 4.00)	4.00 (3.50, 5.50)	>0.05

PER, perampanel; MCD, malformations of cortical development; SeLECTs, self-limiting epilepsy with centrotemporal spikes; SHE, sleep-related hypermotor epilepsy; COVE, childhood occipital visual epilepsy; SeLEAS, self-limited epilepsy with autonomic seizures.

### Effectiveness

**Figure 2:**
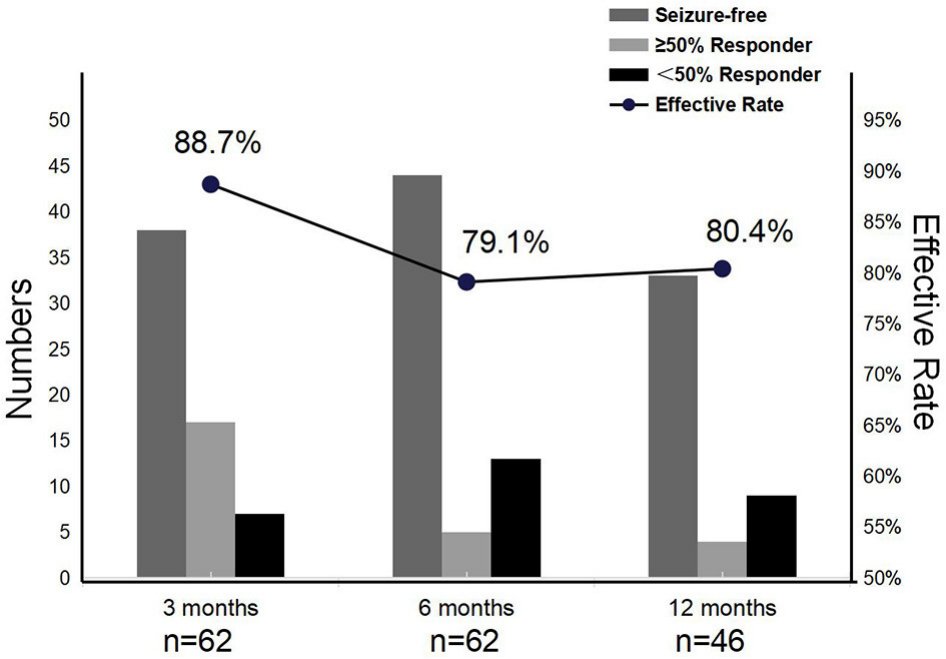
Effectiveness of perampanel at 3-, 6-, and 12-month follow-ups.

We reviewed 62, 62, and 46 patients who remained on PER at 3, 6, and 12 months, respectively. The effective rates of PER treatment at the different time points of evaluation were 88.7% (3 months), 79.1% (6 months), and 80.4% (12 months). In addition, with PER treatment, seizure freedom varied over time, with 61.3% (38/62), 71% (44/62), and 71.7% (33/46) of patients achieving it at the 3-, 6-, and 12-month follow-ups, respectively. The above finding demonstrated the high effectiveness of PER treatment for focal epilepsy in children (Figure 2).

**Figure 3:**
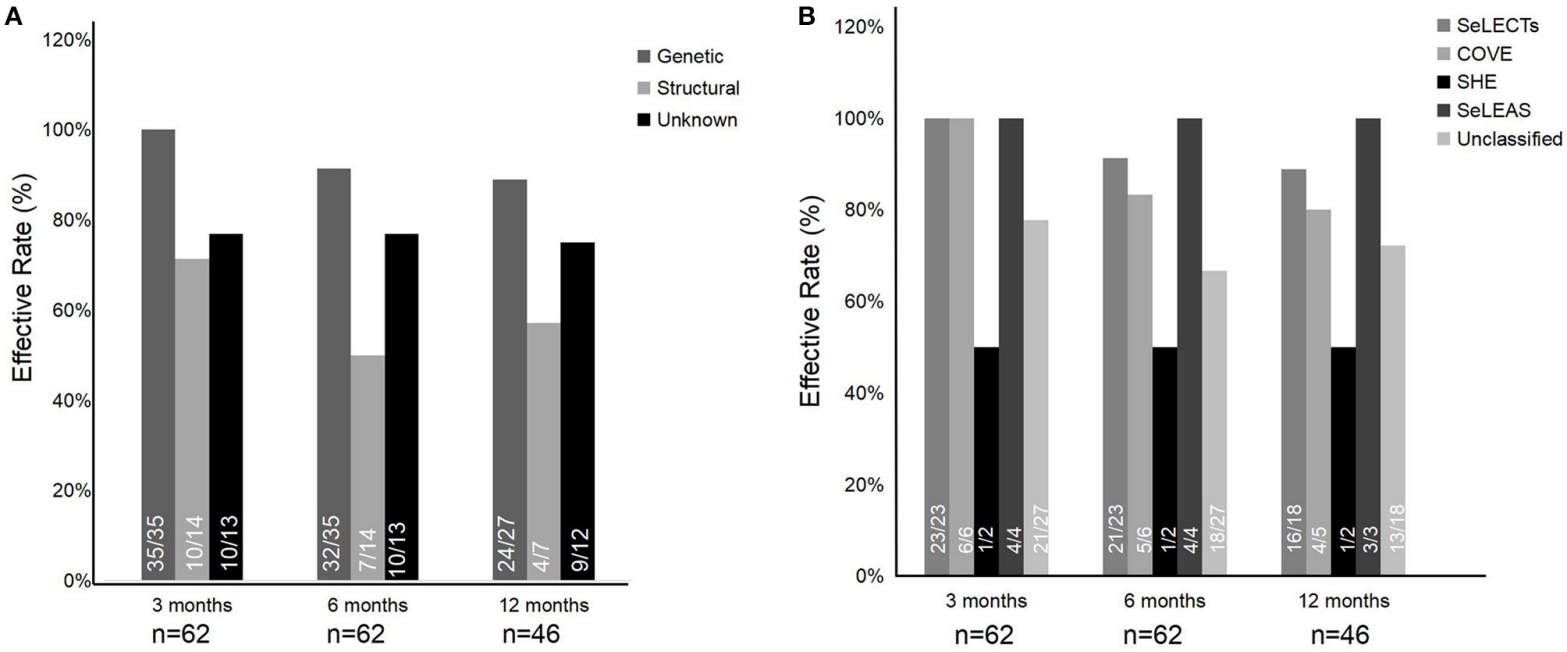
Effective rate among different etiologies **(A)** and epilepsy syndromes **(B)**.

Among the etiologies of epilepsy, the effective rates of genetic etiology, structural etiology, and unknown etiology were generally above 50% at the 3-, 6-, and 12-month follow-ups (Figure 3A). Specifically, the effective rates of genetic etiology descended slightly over time, ranging from 100% (35/35) at the 3-month follow-up to 88.9% (24/27) at the 6-month follow-up. The effective rate of structural etiology was relatively lower but over 50% effective than in the other two etiologies at the 3-, 6-, and 12-month follow-ups. The effective rate of unknown etiology was maintained generally at ~75% at the 3-, 6-, and 12-month follow-ups. In addition, there was no statistical significance among the three etiologies (*p* > 0.05).

Among the epilepsy syndromes, the categories with higher treatment effectiveness were SeLECTs, SeLEAS, and COVE, with an effective rate of above 80%. Two patients were diagnosed with SHE, of whom one was treated effectively by PER at the 3-, 6-, and 12-month follow-ups. In the unknown epilepsy syndromes, the effective rate of patients was more than 60% at the 3-, 6-, and 12-month follow-ups (Figure 3B). Meanwhile, a comparison between self-limited epilepsy (33 patients), including SeLECTs, SeLEAS, and COVE, and non-self-limited epilepsy (29 patients) was also performed. There was no statistical significance of effective rates between self-limited epilepsy and non-self-limited epilepsy in the three terms of follow-up (*p* > 0.05).

### Safety and tolerability

**Figure 4:**
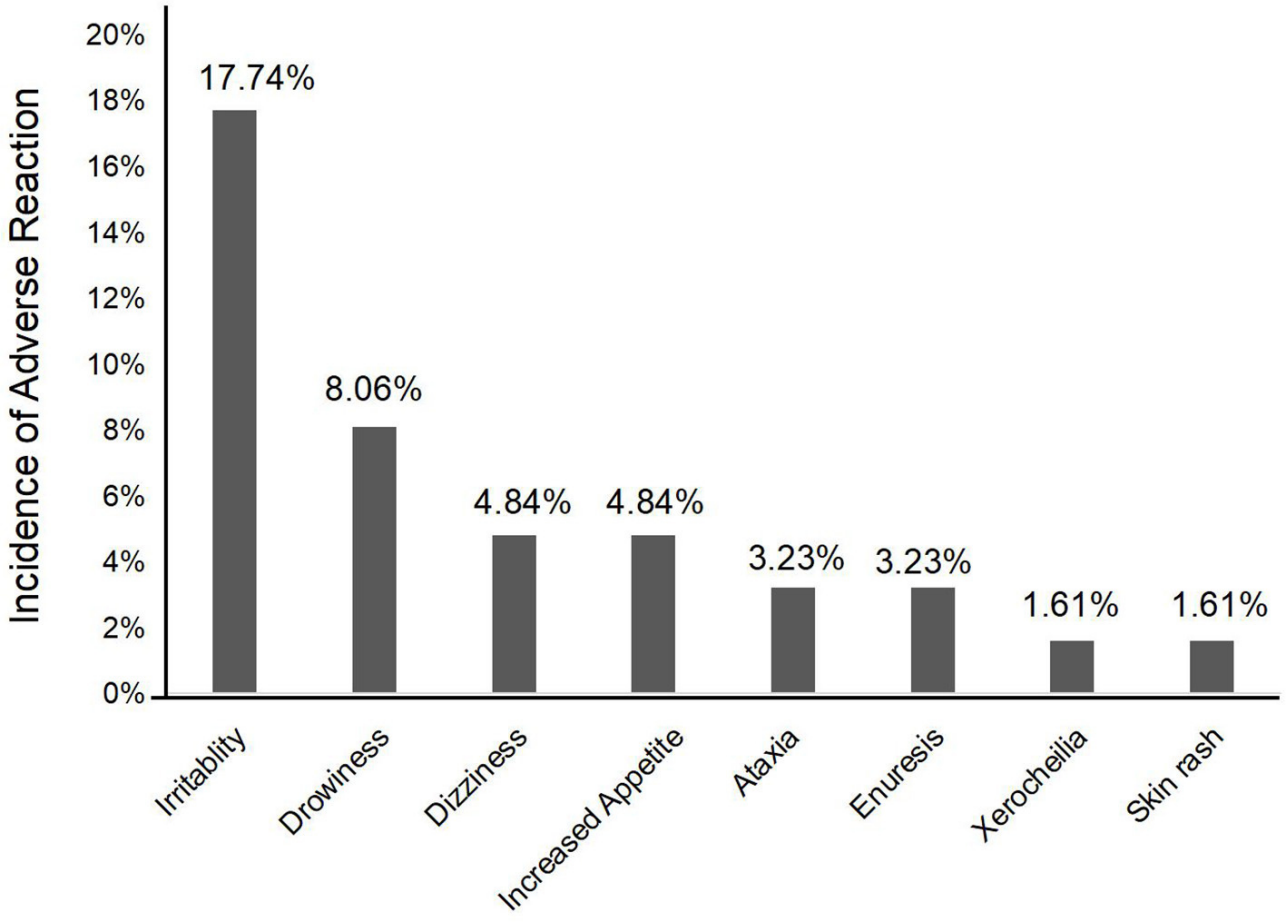
The adverse reactions of perampanel.

A total of 22 pediatric patients (35.5%) experienced at least one adverse reaction (Figure 4), of which irritability (17.7%, *n* = 11) and drowsiness (8.1%, *n* = 5) were the most common, followed by dizziness (4.8%, *n* = 3) and increased appetite (4.8%, *n* = 3). Ataxia (3.2%), enuresis (3.2%), xerocheilia (1.6%), and skin rash (1.6%) were reported in <4% of patients. Among these 22 patients, most adverse reactions were slight and tolerable; only one patient discontinued PER treatment because of skin rash. Meanwhile, these adverse events occurred in patients who started receiving PER treatment, suggesting that the probability of adverse reactions did not correlate with the dosage. All observed adverse events were relatively slight and tolerable over time.

## Discussion

Our study provided potential evidence for the initial monotherapy of PER in the pediatric population with newly diagnosed focal epilepsy. Our retrospective data analysis demonstrated a higher effective rate (~80%) and seizure-free rate (~70%) of PER as initial monotherapy in children with newly diagnosed focal epilepsy at the end of over 6 months of follow-up. Meanwhile, the adverse reactions detected in our study were mild and tolerable, suggesting PER with acceptable safety. To the best of our knowledge, the present study was a novel clinical evaluation of PER treatment in the Chinese pediatric population with focal epilepsy.

In our study, we found that ~70% of patients who were prescribed PER achieved seizure freedom at the end of 6- and 12-month follow-ups, with approximately 80% of patients experiencing ≥50% reduction in seizure frequency, which was consistent with the previous studies. A phase III, open-label study in Japan and South Korea showed that 63.0% of patients (aged ≥12 years) with focal-onset seizures achieved seizure freedom by PER monotherapy on the 4 mg/day dose (15). In a retrospective study of adults from Thailand, the seizure freedom rates of PER monotherapy in newly diagnosed focal-onset epilepsy were 80 and 76% at the end of 6- and 12-month follow-ups, respectively (11). In addition, a multicenter study of PER given as monotherapy indicated that 55% of individuals with focal epilepsy were treated effectively by PER secondary monotherapy (16). These previous studies and our results demonstrated the considerable efficiency of PER monotherapy for focal epilepsy. However, the present evidence focused on the foreign countries and the adult group, since PER was approved for monotherapy use for focal seizures in the United States (11). There was limited information regarding clinical experience with PER monotherapy for Chinese children with newly diagnosed focal epilepsy. Therefore, our findings bridged such a literature gap, broadened our understanding of the application of PER, and encouraged that PER might be useful as an initial monotherapy for focal-onset seizures in pediatric epilepsy.

Our results also showed the high effective rate of PER monotherapy for focal-onset seizures in different epileptic etiologies and epilepsy syndromes at over 6 months of follow-up. Especially, in the etiology of epilepsy, our series showed the effective rates of patients with genetic etiology, structural etiology, and unknown etiology were above 88%, above 50%, and above 75%, respectively. There was no statistical significance in the effectiveness of different etiologies. A retrospective study of children with epilepsy aged from 6 months to 16 years showed that the response rate of PER in epileptic children with structural etiology was 70% (17), which was roughly consistent with our results. The above results suggested that PER monotherapy had generalizability for focal epilepsy with a variety of etiologies in children. In addition, PER monotherapy had higher effective rates for SeLECTs, SeLEAS, and COVE, all of which were more than 80% in our study, which was similar to the previous studies (12). The lower effective rate of SHE was not discussed because of the small number of cases. Nevertheless, in a recent study with a small sample involving children, PER may also have the potential for treating pediatric SHE (12). Future studies need large samples to confirm the effectiveness of PER monotherapy for focal epilepsy in children. Other epilepsy syndromes shown in previous studies, such as Dravet syndrome (8, 12, 13), Lennox–Gastaut syndrome (18–20), and focal epilepsy combined with ESES (10), presented good effectiveness with PER treatment.

In our study, we found that 4 mg/day might be the useful maintenance dose of PER monotherapy for focal-onset seizures in children, although there were a few cases where PER monotherapy at other dosages still proved beneficial. The real-world experiences from Thailand also showed the most common PER dosage was 4 mg/day (61%), and seizure freedom was achieved in 63.0% of patients who were maintained on the 4 mg/day dose (11). This dosage could provide effective seizure reduction and minimize adverse events in most children. According to the recommendation from EMA (21), we formulated the low starting dose based on the children's range of body weight, and then took a slow titration strategy by increasing the daily dose by 1–2 mg every 2 weeks or at even longer intervals. This strategy of PER has been applied to the elderly in previous studies (11), showing good effectiveness and a good safety profile. In addition, a strategy with a low starting dose and a slow titration might be needed for children. Additionally, another favorable advantage of PER was requiring only once-daily dosing, which has a long half-life and could improve the adherence and retention of children, especially school-aged children. A prior study mentioned that the characteristic of a long half-life might be beneficial if a child misses a dose (11).

Overall, PER monotherapy for focal epilepsy had generally good tolerability, with 35.5% of children reporting mild adverse reactions in our study. The most common adverse reactions were irritability and drowsiness, followed by dizziness and increasing appetite, then ataxia, enuresis, xerocheilia, and skin rash. Only one patient discontinued PER monotherapy due to a skin rash. No situation seriously affected the children's vital signs or caused the recurrence and aggravation of epilepsy. Previous studies found that the most common adverse reactions leading to discontinuation in focal-onset seizure studies were dizziness and somnolence (22, 23), particularly in adults or the elderly. Therefore, when somnolence or dizziness occurs, especially in preschool- and school-aged children, taking PER immediately before going to bed is recommended. The most adverse reactions occurred in the titration period, especially when taking a ≤ 4 mg daily dose of PER after starting our study. If the adverse reaction develops during the titration period, the velocity of titration should be slowed down by reducing the dose per addition or prolonged intervals until the adverse reaction resolves.

There are several strengths in our study. It was an advanced exploration of PER treatment for Chinese pediatric patients with newly diagnosed focal epilepsy, showing favorable effectiveness and tolerability. We believe that our results play an essential and instructive role in the clinical practice of PER treatment for focal epilepsy. However, there are potential limitations that should be considered during the interpretation of this study. First, this was a single-center retrospective study without a control group, thus the evidence level was low. Second, the sample size was small, leading to a large gap in sample size between subgroups (such as different etiologies and epilepsy syndromes), which weakened the consistency of conclusions. In addition, the current study might have some selection bias. For example, SeLECTs are characterized by a low seizure frequency, thus its prevalent response in the short term of follow-up after the PER treatment could not confirm the favorable effectiveness of PER entirely. Prospective randomized controlled trials with a larger sample size and long-term follow-up are still needed to verify this conclusion in the future.

## Conclusion

PER as initial monotherapy has favorable effectiveness and tolerability for children with newly diagnosed focal epilepsy. The characteristic of a long half-life allows for once-daily dosing that could promote adherence in children. The current study provides an initial insight into the feasibility of PER monotherapy for focal epilepsy in clinical practice.

## Data availability statement

The raw data supporting the conclusions of this article will be made available by the authors, without undue reservation.

## Ethics statement

The studies involving human participants were reviewed and approved by the Ethics Committee of Jinan Children's Hospital. Written informed consent to participate in this study was provided by the participants' legal guardian/next of kin. Written informed consent was obtained from the individual(s), and minor(s)' legal guardian/next of kin, for the publication of any potentially identifiable images or data included in this article.

## Author contributions

FZ, YR, and GG wrote the first draft. TZ, WH, and HuZ collected the data and fulfilled the data analysis. RJ, JS, and ZG contributed to the conception of the work and revised it critically for important intellectual content. HoZ and YL revised this manuscript and approved it for submission. All authors contributed to the study and approved the submitted version.
